# Leptospirosis under the bibliometrics radar: evidence for a vicious circle of neglect

**DOI:** 10.7189/jogh.09.010302

**Published:** 2019-06

**Authors:** Cyrille Goarant, Mathieu Picardeau, Serge Morand, K Marie McIntyre

**Affiliations:** 1 Institut Pasteur de Nouvelle-Calédonie, Institut Pasteur International Network, Nouméa, New Caledonia; 2 Institut Pasteur, Biology of Spirochetes Unit, French National Reference Centre for Leptospirosis, Paris, France; 3 CNRS ISEM – CIRAD ASTRE, Faculty of Veterinary Technology, Kasetsart University, Bangkok, Thailand; 4 Department of Helminthology, Faculty of Tropical Medicine, Mahidol University, Bangkok, Thailand; 5 Department of Epidemiology and Population Health, Institute of Infection and Global Health, University of Liverpool, Neston, UK; 6 NIHR Health Protection Research Unit in Emerging and Zoonotic Infections, Liverpool, UK

Leptospirosis is a bacterial zoonotic disease that affects over 1 million humans, killing 58 900 every year [1]. It is mostly a disease of vulnerable populations affecting resource-limited people in urban slums and rural settings in developing countries. Leptospirosis also affects livestock and may cost developing economies hundreds of millions of dollars each year and further impoverish subsistence farmers.

Leptospirosis was identified as the only bacterial zoonosis whose impact will likely increase in response to global change in Europe [2], as expected from its numerous environmental drivers [3]. Indeed, recent reports suggest an actual rise in incidence in several European countries, including Germany, France, Croatia and the Netherlands [4].

However, leptospirosis was not considered in a recent systematic study of climate sensitivity of the most important European human and veterinary pathogens [5]. In this systematic study examining climate-sensitivity [5], a prioritization strategy was developed, which used the H-index scores to rank the ‘top’ pathogens’ (rather than diseases’) impact [6]. The H-index prioritisation strategy was validated using correlation between H-indices and global burden estimates for diseases expressed as disability-adjusted life years (DALYs) [7]. Scores for pathogens considered as ‘top pathogens’ ranged between 524 and 57.

We reanalysed the data, evaluating the reason for non-inclusion of leptospirosis. Although a unique disease entity, leptospirosis is caused by several *Leptospira* species; the highest H-index score was for *Leptospira interrogans* (H-index = 45), the name for all virulent leptospires until the 1980s. Leptospirosis was therefore not included in the review, because H-indices for the different virulent *Leptospira* species cannot be treated cumulatively, and no individual species H-index was above the 57 threshold. Re-analysis found that H-indices for “*Leptospira*” or “Leptospirosis” or “*Leptospira* OR leptospirosis” were higher than for single species alone, at 87, 87 and 105, respectively. These indices would have been inside the priority selection window. This omission could be corrected by considering disease entities linked to pathogens within search terms.

The global burden of leptospirosis was recently evaluated by a group of experts (Leptospirosis Epidemiology Research group) convened by World Health Organization (WHO). After extensive data collection and analysis, leptospirosis burden was estimated at 2.90 million DALYs [8]. If included in the correlation (**Figure 1**, panel A), it falls well below the regression line, providing evidence of insufficient research attention in relation to burden, the hallmark of neglect. Yet, leptospirosis is not included in the WHO list of neglected tropical diseases. Among the consequences identified, leptospirosis research is significantly under-resourced; the research area is unattractive to both funders (**Figure 1**, panel B, funding data from G-finder, see Table S1 in **Online Supplementary Document**) and researchers and the leptospirosis research community is shrinking due to lack of visibility and insufficient resources. In practical terms, no significant progress has been made to develop major areas beyond a leptospirosis vaccine [9] and the time-consuming diagnostic reference test for serology [10]; both were developed a century ago.

**Figure 1:**
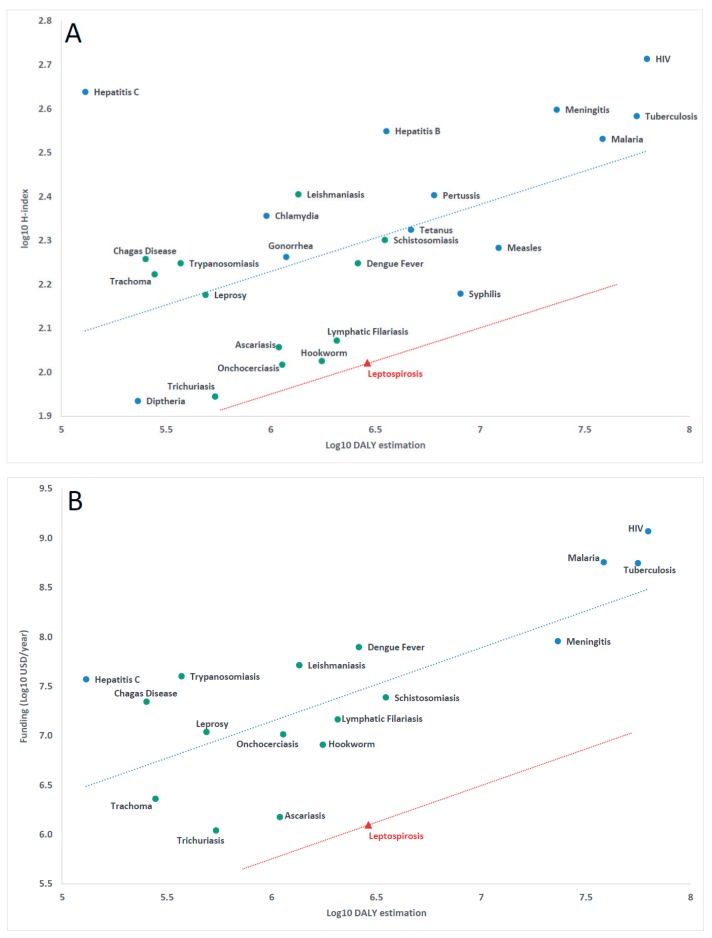
Relationship between the global burden of disease (in DALYs estimated by World Health Organization (WHO) for 2015) and relative research impact (H-index score, calculated using Web of Knowledge and a search period from 1900 to 2018). This provides evidence for the neglect of leptospirosis in panel A. Relationship between the global burden of disease and research funding (annual funding in USD adjusted to 2016 US$, from G-finder) in panel B, confirming the negative impact on research resources in relation to leptospirosis burden. Green dots are for diseases classified as “neglected tropical diseases” by WHO, a red triangle defines leptospirosis; blue dots are for other diseases. Parallel to the linear regression line calculated using information for all diseases in each plot (blue), the red line illustrates the distance below the regression line for leptospirosis. Detailed data are available in Table S1 in **Online Supplementary Document**.

**Figure:**
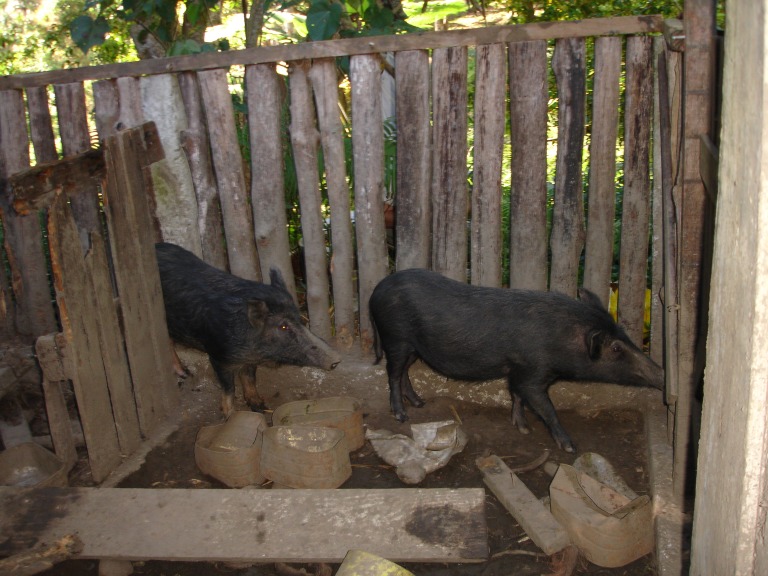
Photo: Backyard subsistence pig farming is probably a major source of leptospirosis in most of the Pacific Island Countries and Territories, where the highest burden is reported from around the globe (from C. Goarant, used with permission)

Leptospirosis, a severe, potentially fatal disease lays at the crossroads of several Sustainable Development Goals. It continues to inflict its high burden on the most vulnerable populations; especially those with least health infrastructure in the developing world, amidst almost total indifference. Breaking the vicious circle of neglect is urgent.

## Additional Material

Online Supplementary Document
